# Supplementary material for: Nitrogen cycling activities during decreased stratification in the coastal oxygen minimum zone off Namibia
Source: Front Microbiol. 2023 Feb 10;14:1101902. doi: 10.3389/fmicb.2023.1101902 (PMC9950273; doi:10.3389/fmicb.2023.1101902)
Supplement: Supplementary file 1 [file Data_Sheet_1.PDF]

Supplementary Material

1 Supplementary Figures and Tables

1.1 Supplementary Figures

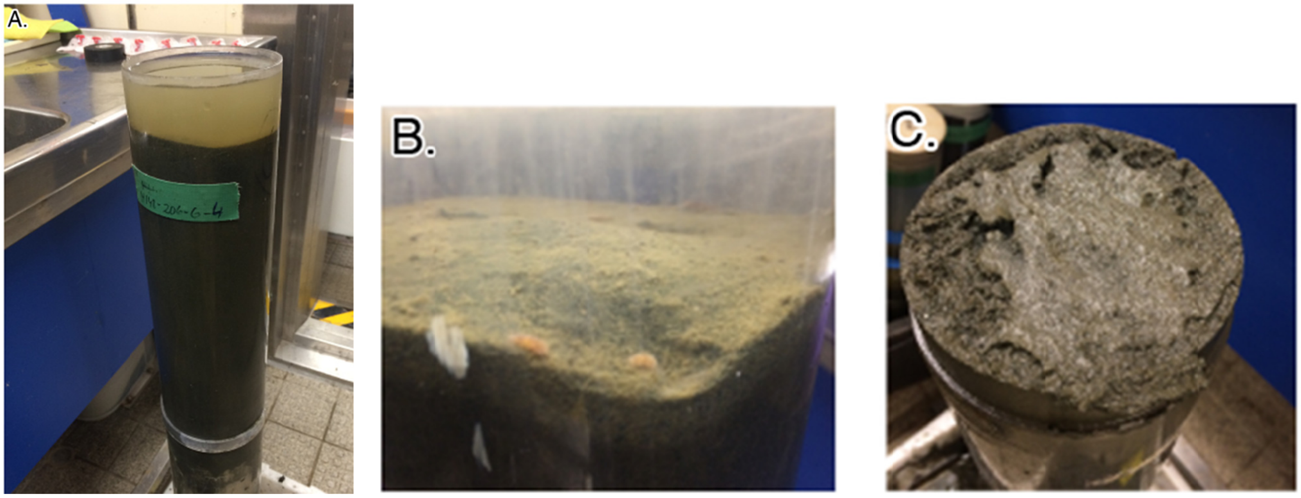

Supplementary Figure 1. Sediment cores retrieved from the Namibian shelf. (**A**) Pristine core recovered, together with bottom water. (**B**) Zoom in on the core top surface evidencing an undisturbed sediment-water interface. (**C**) Photo of core inside at ca. 15 cm depth, taken during sectioning. The sediment is composed primarily of green mud and foraminiferal sand. Photos are reproduced from (Orsi et al., 2020b, 2020a).

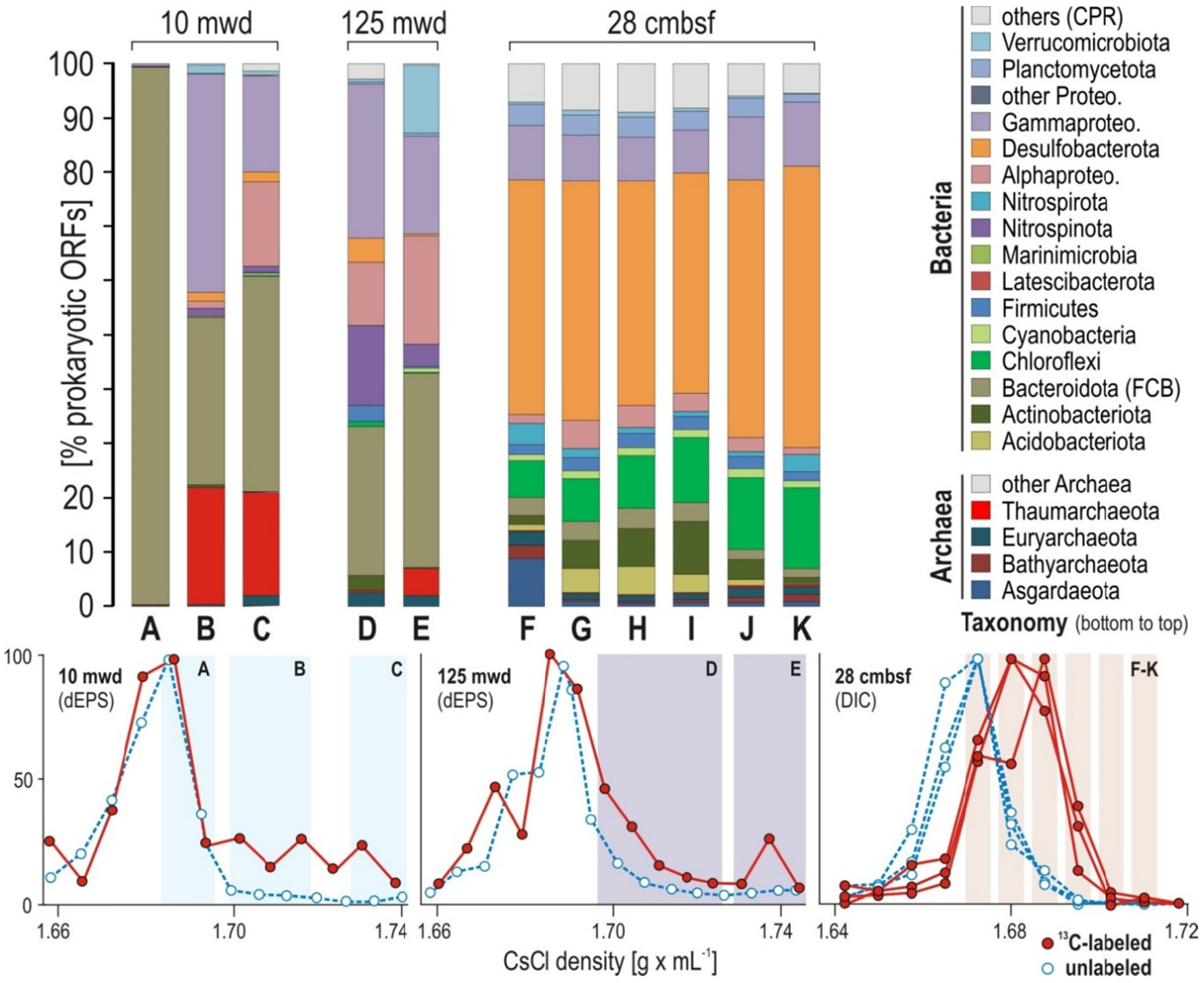

Supplementary Figure 2. Taxonomic assemblages from the total ^{13}C-labeled "isotopically heavy" DNA fractions identified via qPCR buoyant density curves. (**Top**) Taxonomic assemblages of the "heavy" DNA fractions [% prokaryotic ORFs] considered indicative of phyla showing ^{13}C-dEPS and ^{13}C-bicarbonate assimilation. (**Bottom**) qPCR buoyant density curves for 16S rRNA genes obtained from DNA fractions of the 18 hours SIP incubations with water from 10 and 125 mwd and ^{13}C-dEPS, and 10 days SIP incubations with sediment from 28 cmblf at site 6 and ^{13}C-bicarbonate (red dots: ^{13}C-labeled; blue dots: unlabeled controls). Illustration is modified after (Vuillemin et al., 2022).

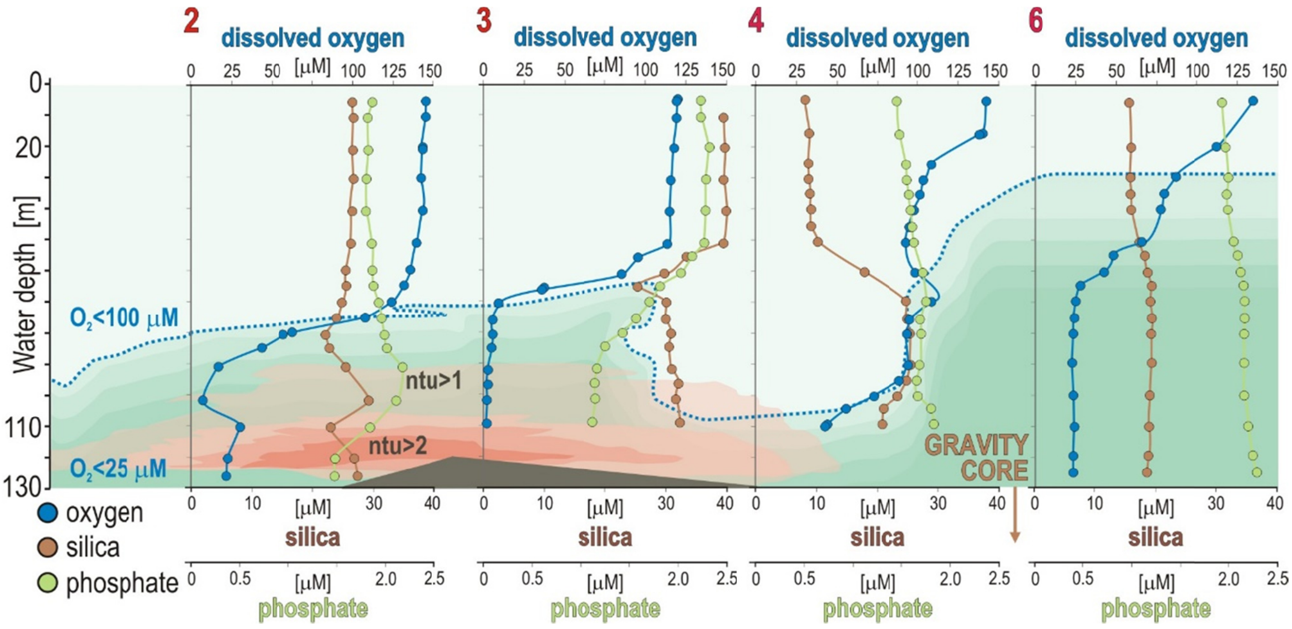

Supplementary Figure 3. Geochemical profiles for the water column at site 2, 3, 4 and 6. Concentration profiles for dissolved oxygen (blue) with an oxycline defined as <100 µM (dotted line), silica (brown), phosphate (green) and turbidity (pink) in nephelometric turbidity units (ntu) at each successive sampling site. Water column data are from (Siccha and Kucera, 2018; Ferdelman et al., 2021a; Garaba et al., 2021).

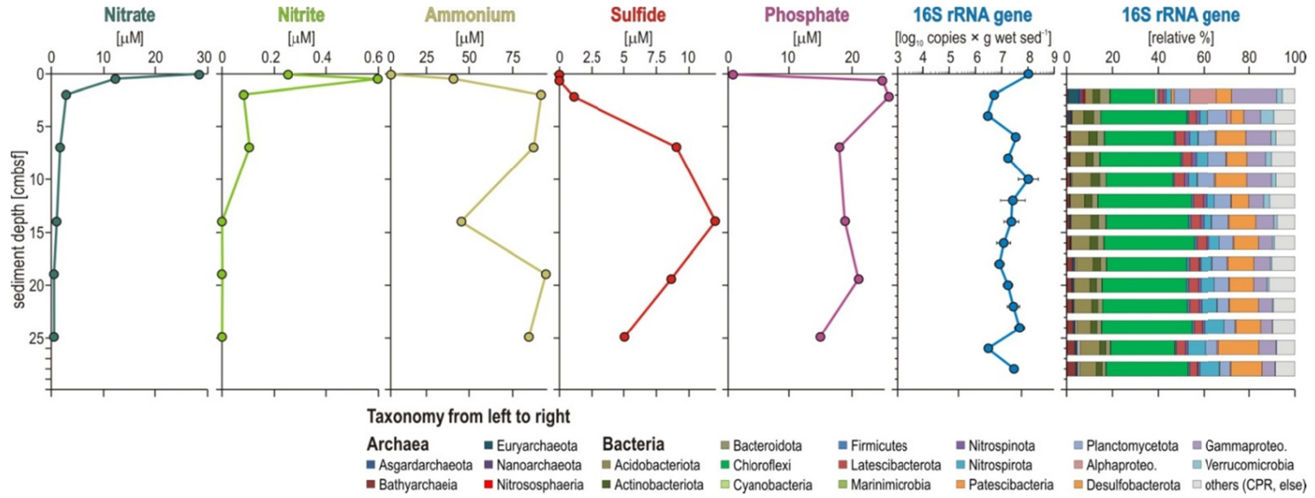

Supplementary Figure 4. Geochemical profiles for the sediment at site 6 with 16S rRNA gene density and diversity. From left to right: Geochemical profiles of nitrate, nitrite, ammonium, sulfide and phosphate [μM], followed by 16S rRNA gene density [$\log_{10}$ gene copies × g wet sed^{-1}] assessed via quantitative PCR and 16S rRNA gene taxonomic diversity [relative %]. Pore water data are from (Ferdelman et al., 2021b).

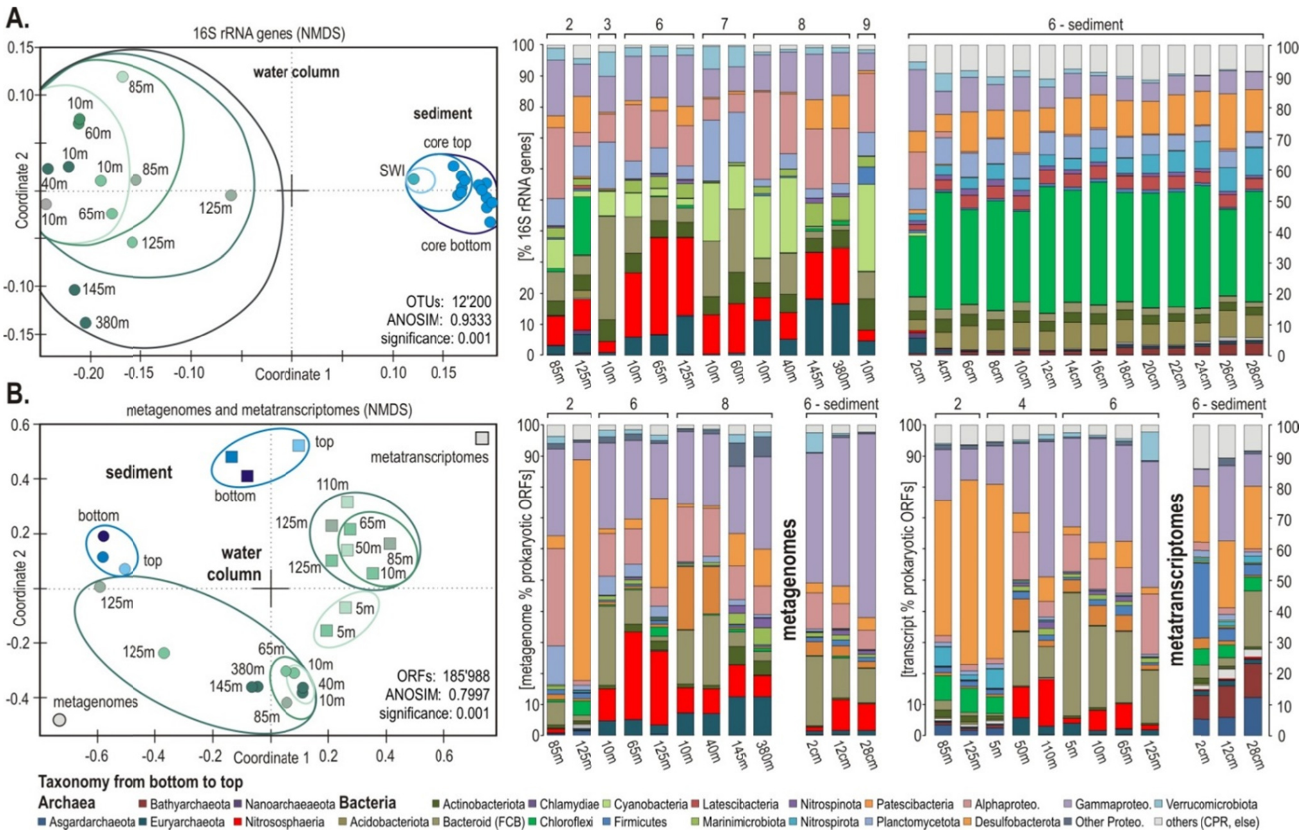

Supplementary Figure 5. Beta diversity and taxonomic assemblages of 16S rRNA gene amplicons, metagenomes and metatransciptomes. (**A**) Non-metric multidimensional scaling (NMDS) plot based on all OTUs across all water column and sediment samples and their corresponding taxonomic assemblages [% 16S rRNA genes]. (**B**) NMDS plot based on all prokaryotic ORFs from the metagenomes (circles) and metatranscriptomes (squares) and their corresponding taxonomic assignments [% ORFs]. Illustration is modified from (Vuillemin et al., 2022).

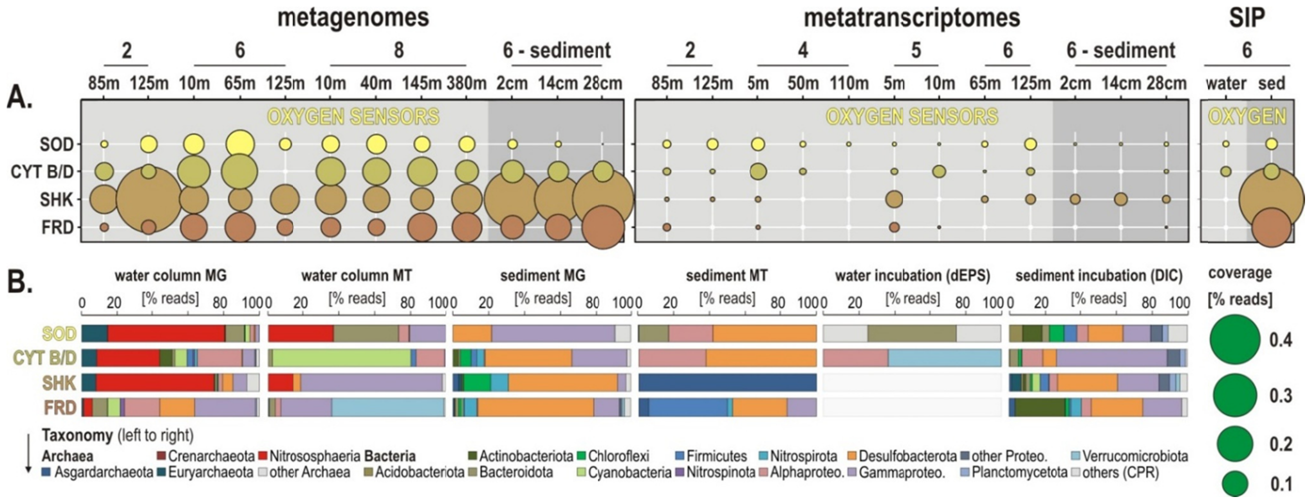

Supplementary Figure 6. Metabolic functions and activities related to putative oxygen sensors in the water column, sediment and SIP incubations, and the corresponding taxonomic assignments at the phylum level. (A) Bubble plot showing the relative potential and expression level of metabolic functions [% total reads] assigned to marker genes encoding putative oxygen sensors in the metagenomes, metatranscriptomes, stable-isotope probing (SIP) water and sediment incubations (left to right). (**B**) Taxonomic bar charts [% reads] for the corresponding marker genes at the phylum level in the metagenomes (MG), metatranscriptomes (MT) and SIP incubations with water and ^{13}C-labeled dEPS and with sediment and ^{13}C-labeled bicarbonate (DIC).

Abbreviations: SOD: superoxide dismutase / CYT B/D: cytochromes cbb3, bd2 and b / SHK: sensor histidine kinase / FRD: fumarate reductase.

1.2 Supplementary Tables

Supplementary Table 1. Sequencing and assembly statistics for metatranscriptomes, metagenomes, and SIP-metagenomes.

METATRANSCRIPTOMES								
**Station**	**Sample type**	**oxygen level**	**Depth (m)**	**Replicate**	**# reads (millions)**	**# contigs**	**reads mapping (millions)**	**% of reads mapping to contigs**
**site-202**	water column	oxycline	85	a	3	13,274	2.1	70.0
				b	3.6	27,862	2.2	61.1
		OMZ	125	a	4.7	19,254	3.5	74.5
				b	3.4	15,817	2.5	73.5
**site-204**	water column	Surface (oxic)	5	a	10.2	67,010	8.2	80.4
				b	9.1	41,408	7	76.9
		oxycline	50	a	9.4	21,960	8.2	87.2
				b	7.6	17,647	6.6	86.8
		OMZ	110	a	13.8	27,168	12.2	88.4
				b	7.9	29,864	6.2	78.5
**site-206**	water column	oxic	5	a	5.7	19,334	4.6	80.7
				b	6.7	37,547	5.1	76.1
			10	a	7.2	20,735	6.3	87.5
				b	3.7	10,464	3.2	86.5
		oxycline	65	a	7.2	16,298	6.1	84.7
				b	3.5	9,860	2.7	77.1
		OMZ	125	a	1.7	5,526	1.3	76.5
				b	5.3	16,420	4.2	79.2
				c	7.7	25,788	6.1	79.2
	sediments	hypoxic	core top	a	4.6	2,602	3.7	80.4
				b	11.1	2,927	9.2	82.9
		sulfidic	12 cm	a	3.8	4,362	2.7	71.1
				b	2.2	2,726	1.3	59.1
				c	3.4	5,888	2.1	61.8
			28 cm	a	3.8	7,429	2.3	60.5
				b	5.7	9,636	4.2	73.7
				c	4.1	5,660	2.7	65.9

METAGENOMES								
**Station**	**Sample type**	**oxygen level**	**Depth (m)**	**Replicate**	**# reads (millions)**	**# contigs**	**reads mapping (millions)**	**% of reads mapping to contigs**
**site-202**	water column	oxycline	85	a	5.5	79,040	1.7	30.9
		OMZ	125	a	3.6	81,412	0.6	16.7
**site-206**	water column	oxic	10	a	4.9	102,742	1.5	30.6
		oxycline	65	a	5.8	95,018	1.2	20.7
		OMZ	125	a	7.1	49,885	0.88	12.4
	sediments	hypoxic	core top	a	15.7	80,933	1.26	8.0
		sulfidic	12 cm	a	5.1	12,580	0.15	2.9
			28 cm	a	3.6	9,583	0.12	3.3
site-208	water column	oxic	10	a	5	70,549	1.2	24.0
			145	a	7.8	125,658	1.9	24.4
			380	a	5	79,761	1.4	28.0

SIP-metagenomes							
**Station**	**SIP incubation**	**Sample type**	**oxygen level**	**Depth (m)**	**^{13}C-SIP metagenome**	**Density range (g mL^{-1})**	**# reads (millions)**
**site-206**	^{13}C-dEPS	water column	oxic	10m	10m_a	1.685-1.70	4.9
					10m_b	1.70-1.74	4.7
					10m_c	1.74-1.76	3.7
			OMZ	125m	125m_a	1.70-1.73	5.8
					125m_b	1.73-1.745	2.7
	^{13}C-bicarbonate	sediment	sediment	23 cm	23cm_a	1.682	9.6
					23cm_b	1.689	2.7
					23cm_c	1.697	15.5
					23cm_d	1.705	12.8
					23cm_e	1.713	8.7
					23cm_f	1.722	1.6

Supplementary Table S2. Binning statistics for metatranscriptomes.

Sample	total_num_reads	num_INDELs_reported	total_reads_kept	num_SNVs_reported	total_reads_mapped	percent_mapped
Site 202 85m (MT)	5994376	7390	2543311	163023	2543311	42.43
Site 202 125m (MT)	6646454	10235	3257803	245540	3257803	49.02
Site 204 5m (MT)	17164574	12126	8786590	259866	8786590	51.19
Site 204 50m (MT)	15809144	13804	8992028	321241	8992028	56.88
Site 204 110m (MT)	21100648	9460	15912601	161755	15912601	75.41
Site 206 5m (MT)	11629756	8151	5088200	139498	5088200	43.75
Site 206 10m (MT)	11399926	14046	5529901	320131	5529901	48.51
Site 206 65m (MT)	9887270	11220	5402852	249057	5402852	54.64
Site 206 125m (MT)	20230136	13643	10912948	344857	10912948	53.94
Site 206 core top3 (MT)	3393648	7991	1706512	111746	1706512	50.29
Site 206 core top (MT)	9222134	12171	4416491	172450	4416491	47.89
Site 206 core 12cm (MT)	8446572	9040	2695838	213050	2695838	31.92
Site 206 28cm (MT)	59101508	17092	6830510	943736	6830510	11.56

bins	total_length	num_contigs	N50	GC_content	percent_completion	percent_redundancy	t_domain	t_phylum	t_class	t_order	t_family	t_genus	t_species
MAXBIN__031	6449290	3961	1549	52.0	91.55	67.61	Bacteria	Desulfobacterota	Syntrophobacteria	BM002	BM002	BM002	BM002 sp002899795
MAXBIN__034	6583881	4089	1513	63.6	87.32	88.73	Bacteria	Actinobacteriota	Acidimicrobiia	UBA5794	UBA4744	UBA4744	UBA4744 sp002403855
MAXBIN__036	3542706	2068	1711	64.1	67.61	32.39	Bacteria	Myxococcota					
MAXBIN__033	2915624	1980	1428	61.7	63.38	35.21	Bacteria	Myxococcota	Polyangia	Polyangiales	SG8-38	SG8-38	SG8-38 sp003647035
MAXBIN__019	738826	497	1426	47.0	60.56	92.96	Bacteria						
MAXBIN__003	2410097	1526	1558	45.3	54.93	21.13	Bacteria	Proteobacteria	Gammaproteobacteria	Enterobacterales	Alteromonadaceae	Pseudoalteromonas	
MAXBIN__009	434046	258	1616	41.1	50.70	53.52	Bacteria	Proteobacteria	Gammaproteobacteria	PS1	Thioglobaceae	Thioglobus	Thioglobus singularis
MAXBIN__018	102837	66	1513	32.6	50.70	36.62	Bacteria	Bacteroidota	Bacteroidia	Flavobacteriales	Flavobacteriaceae	MAG-121220-bin8	MAG-121220-bin8 sp004214185
MAXBIN__032	2237305	1428	1537	46.9	47.89	11.27	Bacteria	Desulfobacterota	Syntrophobacteria	BM002	BM002	BM002	BM002 sp002899795
MAXBIN__025	489338	327	1400	30.7	47.89	43.66	Bacteria	Bacteroidota	Bacteroidia	Flavobacteriales			
MAXBIN__035	2493781	1879	1276	69.4	42.25	19.72	Bacteria	Myxococcota	UBA9160	UBA9160	UBA6930	UBA6930	UBA6930 sp002450755
MAXBIN__024	233551	157	1386	43.5	42.25	23.94	Bacteria						
MAXBIN__007	267590	156	1683	32.2	39.44	4.23							
MAXBIN__021	1617878	1099	1373	39.7	38.03	35.21	Bacteria	Proteobacteria	Gammaproteobacteria	PS1	Thioglobaceae	Thioglobus	Thioglobus sp001628405
MAXBIN__026	1308179	908	1368	42.4	36.62	22.54							
MAXBIN__011	106535	48	2371	45.0	32.39	4.23	Bacteria	Proteobacteria	Gammaproteobacteria	Pseudomonadales	Porticoccaceae	HTCC2207	
MAXBIN__013	944322	704	1286	49.2	30.99	11.27	Bacteria	Proteobacteria	Gammaproteobacteria	Pseudomonadales			
MAXBIN__016	222421	166	1293	37.2	29.58	22.54	Bacteria	SAR324	SAR324	SAR324	NAC60-12	Arctic96AD-7	Arctic96AD-7 sp002685535
MAXBIN__022	133005	86	1570	38.4	28.17	19.72	Bacteria						
MAXBIN__014	177509	132	1304	32.9	26.76	28.17	Bacteria	Bacteroidota	Bacteroidia	Flavobacteriales	Flavobacteriaceae	MAG-121220-bin8	MAG-121220-bin8 sp002700465
MAXBIN__023	307720	215	1307	37.5	23.94	14.08	Bacteria						
MAXBIN__015	102878	67	1501	41.1	23.94	4.23	Bacteria	Verrucomicrobiota	Lentisphaeria	Lentisphaerales	Lentisphaeraceae	Lentisphaera	Lentisphaera araneosa
MAXBIN__038	1660543	1282	1246	67.7	22.54	9.86							
MAXBIN__010	993817	673	1409	40.5	22.54	21.13	Bacteria	Proteobacteria	Gammaproteobacteria	PS1	Thioglobaceae	Thioglobus	Thioglobus singularis
MAXBIN__006	838719	546	1501	41.4	22.54	8.45	Bacteria						
MAXBIN__004	123978	69	1799	35.6	21.13	1.41	Bacteria	Marinisomatota	Marinisomatia	Marinisomatales	TCS55	TCS55	TCS55 sp001577025
MAXBIN__037	1869842	1462	1221	66.9	0.00	0.00							
MAXBIN__008	1481191	950	1536	42.0	0.00	0.00							
MAXBIN__029	733196	440	1500	35.9	0.00	0.00	Bacteria						
MAXBIN__002	668219	440	1498	46.9	0.00	0.00	Bacteria	Proteobacteria	Gammaproteobacteria	Enterobacterales	Vibrionaceae	Aliivibrio	Aliivibrio salmonicida
MAXBIN__028	635327	444	1369	47.3	0.00	0.00	Bacteria						
MAXBIN__017	499776	310	1538	35.0	0.00	0.00	Bacteria	Bacteroidota	Bacteroidia	Flavobacteriales	BACL11	UBA8444	UBA8444 sp003454845
MAXBIN__001	248620	152	1585	46.4	0.00	0.00							
MAXBIN__012	239179	165	1417	31.5	0.00	0.00							
MAXBIN__005	207317	135	1573	36.4	0.00	0.00	Bacteria	Proteobacteria	Gammaproteobacteria	PS1	Thioglobaceae	Thioglobus	Thioglobus singularis
MAXBIN__027	155301	122	1174	34.0	0.00	0.00							
MAXBIN__020	121882	81	1527	31.4	0.00	0.00	Bacteria	Bacteroidota	Bacteroidia	Flavobacteriales	Flavobacteriaceae	MED-G11	
MAXBIN__030	102295	53	1953	33.1	0.00	0.00	Bacteria	Bacteroidota	Bacteroidia	Flavobacteriales	Flavobacteriaceae	Maribacter	Maribacter sp000153165

1.3 Supplementary References

Ferdelman, T. G., Klockgether, G., Imhoff, K., and Mohrholz, V. (2021a). Meteor Expedition M148/2 EreBUS nutrient data from Benguela Upwelling System and Angola Gyre. doi: 10.1594/PANGAEA.931090.

Ferdelman, T. G., Klockgether, G., Imhoff, K., and Gomez-Saez, G. V. (2021b). Meteor Expedition M18/2 EreBUS sediment porewater nutrient and sulfur from station M148/2_206-6. doi: 10.1594/PANGAEA.931097.

Garaba, S. P., Thölen, C., Ferdelman, T. G., and Zielinski, O. (2021). Optical water quality variables (Secchi disk depth and Forel-Ule colour indexes) observed during cruise M148/2 aboard RV METEOR. doi: 10.1594/PANGAEA.928943.

Orsi, W. D., Morard, R., Vuillemin, A., Eitel, M., Wörheide, G., Milucka, J., et al. (2020a). Anaerobic metabolism of Foraminifera thriving below the seafloor. *ISME J.* 14, 2580–2594. doi: 10.1038/s41396-020-0708-1.

Orsi, W. D., Vuillemin, A., Rodriguez, P., Coskun, Ö. K., Gomez-Saez, G. V., Lavik, G., et al. (2020b). Metabolic activity analyses demonstrate that Lokiarchaeon exhibits homoacetogenesis in sulfidic marine sediments. *Nat. Microbiol.* 5, 248–255. doi: 10.1038/s41564-019-0630-3.

Siccha, M., and Kucera, M. (2018). Processed multinet CTD data from METEOR cruise M148/2. doi: 10.1594/PANGAEA.895640.

Vuillemin, A., Coskun, Ö. K., and Orsi, W. D. (2022) Microbial Activities and Selection from Surface Ocean to Subseafloor on the Namibian Continental Shelf. *Appl. Environ. Microbiol.* 88, e00216-22. doi: 10.1128/aem.00216-22.
